# Physicochemical Characterization and Biological Properties of Pine Honey Produced across Greece

**DOI:** 10.3390/foods11070943

**Published:** 2022-03-25

**Authors:** Eleni Tsavea, Fotini-Paraskevi Vardaka, Elisavet Savvidaki, Abdessamie Kellil, Dimitrios Kanelis, Marcela Bucekova, Spyros Grigorakis, Jana Godocikova, Panagiota Gotsiou, Maria Dimou, Sophia Loupassaki, Ilektra Remoundou, Christina Tsadila, Tilemachos G. Dimitriou, Juraj Majtan, Chrysoula Tananaki, Eleftherios Alissandrakis, Dimitris Mossialos

**Affiliations:** 1 Laboratory of Microbial Biotechnology–Molecular Bacteriology–Virology, Department of Biochemistry & Biotechnology, University of Thessaly, Biopolis, 41500 Larissa, Greece; elenats89@hotmail.com (E.T.); tsadila@bio.uth.gr (C.T.); tidimitr@bio.uth.gr (T.G.D.); 2 Laboratory of Quality and Safety of Agricultural Products, Landscape and Environment, Department of Agriculture, Hellenic Mediterranean University, Stavromenos PC, 71410 Heraklion, Greece; foteinivardak@hmu.gr (F.-P.V.); elisavvidaki@hmu.gr (E.S.); 3 Food Quality & Chemistry of Natural Products, Mediterranean Agronomic Institute of Chania, International Centre for Advanced Mediterranean Agronomic Studies, 73100 Chania, Greece; kellilabdessamie@gmail.com (A.K.); grigorakis@maich.gr (S.G.); yiota@maich.gr (P.G.); sofia@maich.gr (S.L.); hlektra@maich.gr (I.R.); 4 Laboratory of Apiculture-Sericulture, Faculty of Agriculture, Forestry and Natural Environment, School of Agriculture, Aristotle University of Thessaloniki, 54124 Thessaloniki, Greece; dkanelis@agro.auth.gr (D.K.); mdimou@agro.auth.gr (M.D.); tananaki@agro.auth.gr (C.T.); 5 Laboratory of Apidology and Apitherapy, Department of Molecular Genetics, Institute of Molecular Biology, Slovak Academy of Sciences, Dubravska Cesta 21, 845 51 Bratislava, Slovakia; marcela.bucekova@savba.sk (M.B.); jana.godocikova@savba.sk (J.G.); juraj.majtan@savba.sk (J.M.); 6 Department of Microbiology, Faculty of Medicine, Slovak Medical University, Limbova 12, 833 03 Bratislava, Slovakia; 7 Institute of Agri-Food and Life Sciences Agro-Health, Hellenic Mediterranean University Research Center, Stavromenos PC, 71410 Heraklion, Greece

**Keywords:** honeydew, pine honey, physicochemical parameters, melissopalynology, sensory evaluation, bioactivity, antimicrobial, antioxidant

## Abstract

Pine honey is a honeydew honey produced in the East Mediterranean region (Greece and Turkey) from the secretions of the plant sucking insect *Marchalina hellenica* (Gennadius) (Coccoidea: Marchalini-dae) feeding on living parts of *Pinus* species. Nowadays, honeydew honey has attracted great attention due to its biological activities. The aim of this study was to study unifloral pine honey samples produced in Greece regarding their physicochemical parameters and antioxidant and antibacterial activity against five nosocomial and foodborne pathogens. These honeys showed physicochemical and microscopic characteristics within the legal limits, except for diastase activity, a parameter known to be highly variable, depending on various factors. Substantially higher levels of H_2_O_2_ were estimated compared to other types of honeydew honey, whereas protein content was similar. The total phenolic content was 451.38 ± 120.38 mg GAE/kg and antiradical activity ranged from 42.43 to 79.33%, while FRAP values (1.87 to 9.43 mmol Fe^+2^/kg) were in general higher than those reported in the literature. Various correlations could be identified among these parameters. This is the first attempt to investigate in depth the antibacterial activity of pine honey from Greece and correlate it with honey quality parameters. All tested honeys exerted variable but significant antibacterial activity, expressed as MIC and MBC values, comparable or even superior to manuka honey for some tested samples. Although honey antibacterial activity is mainly attributed to hydrogen peroxide and proteins in some cases (demonstrated by elevated MICs after catalase and Proteinase K treatment, respectively), no strong correlation between the antibacterial activity and hydrogen peroxide concentration or total protein content was demonstrated in this study. However, there was a statistically significant correlation of moisture, antioxidant and antibacterial activity against *Klebsiella pneuomoniae*, as well as antioxidant and antibacterial activity against *Salmonella* ser. Typhimurium. Interestingly, a statistically significant negative correlation has been observed between diastase activity and *Staphylococcus aureus* antibacterial activity. Overall, our data indicate multiple mechanisms of antibacterial activity exerted by pine honey.

## 1. Introduction

Honey is a sweet, supersaturated solution of carbohydrates, composed of glucose, fructose, oligo- and polysaccharides, water, and other substances, such as proteins, enzymes, vitamins, minerals, phenolic compounds and amino acids that are of nutritional and health significance [1]. The chemical composition of honey and physicochemical parameters are variable and related to the botanical origin, geographic area and environmental conditions [2,3].

Pine honey is a honeydew honey produced by honeybees from the secretions of the plant sucking insect *Marchalina hellenica* (Gennadius) (Coccoidea: Marchalinidae) feeding on living parts of *Pinus* species. It is a typical honeydew honey, with high ash content, pH value and electrical conductivity. Additionally, the fructose and glucose content are low; therefore, its tendency to crystallize is low. This type of honey is produced in the Mediterranean region and specifically in Greece and Turkey. It constitutes 60–65% and 50% of the total annual honey production in Greece and Turkey, respectively [4]. It is of high nutritional value due to, among others, its high content of minerals such as potassium, calcium, iron, phosphorus, magnesium, sodium and zinc [5].

Greek legislation has adopted the Council Directive 2001/110/EC [6] regarding the physicochemical properties of honey. In addition, the Government Gazette B-239/23–2-2005 [7] describes the required properties for monofloral Greek honey varieties, according to which the electrical conductivity of Greek pine honey must be higher than 0.9, while also the presence of honeydew elements must be significant.

In recent decades, scientific interest has been focused on the antibacterial activity of diverse types of honey against clinical and foodborne pathogens [8,9,10,11,12,13,14,15,16] as well as on antioxidant activity [12,17,18,19,20]. Recently, honey antibacterial activity has been proposed as a valuable parameter determining its quality which takes into account the biological properties of honey [21].

Hydrogen peroxide (H_2_O_2_) is often considered as the major antibacterial compound of honey and it is produced by the enzyme glucose oxidase, which converts glucose into gluconic acid [22]. Several studies have clearly demonstrated the strong correlation between the antibacterial activity and the presence of H_2_O_2_ in certain types of blossom honeys [23,24,25,26,27]. However, a recent study by Farkasovska et al. [25] reported a weak or no correlation between antibacterial activity against particular bacteria and H_2_O_2_ concentration in linden honey samples. Similarly, despite the high level of H_2_O_2_ measured in Slovak honeydew honeys, no significant correlation was found between their overall antibacterial activity and the level of H_2_O_2_ [28]. In addition to H_2_O_2_, peptides and proteins such as bee defensin-1 and MRJP glycoproteins have been isolated from various honeys exhibiting antibacterial activity through cell lysis [29,30]. Additionally, phytochemicals such as phenolic compounds may significantly contribute to the antibacterial and antioxidant activity of honey, in particular honeydew honey [31].

A small fraction of honey’s composition (2–5% of honey dry weight) contains compounds responsible for a plethora of biological properties, such as anti-inflammatory, antimicrobial antimutagenic, antioxidant, antiproliferative and antithrombotic [32,33]. The antioxidant activity involves the deactivation of free radicals, and it is classified into two mechanisms: hydrogen atom transfer (HAT) and electron transfer (ET). In the former, a hydrogen atom is donated to the radical, while in the latter, a single electron is transferred [34]. Phenolics are a class of phytochemicals that are primarily responsible for the antioxidant activity exerted by honey. Other non-phenolic compounds with the same activity are enzymes (catalase and peroxidase), ascorbic acid and carotenoids. Phenolic compounds that have been reported in honey include phenolic acids (coumaric, caffeic, ellagic, ferulic and chlorogenic acids) and flavonoids (chrysin, kaempferol, pinosembrin, quercetin, galangin, hesperetin, and myricetin) [35]. Very often, the presence of these compounds is expressed as total phenolic content, and it is positively correlated with the antioxidant capacity as well as antibacterial activity of the tested honey [27,34].

The aim of this study was to characterize unifloral pine honeys produced in Greece regarding their physicochemical parameters, antioxidant activity as well as their antibacterial activity against nosocomial and foodborne pathogens. To the best of our knowledge, this is the first in-depth attempt to investigate the antibacterial activity of pine honey from Greece and correlate it with various honey properties.

## 2. Materials and Methods

### 2.1. Honey Samples

**Figure 1:**
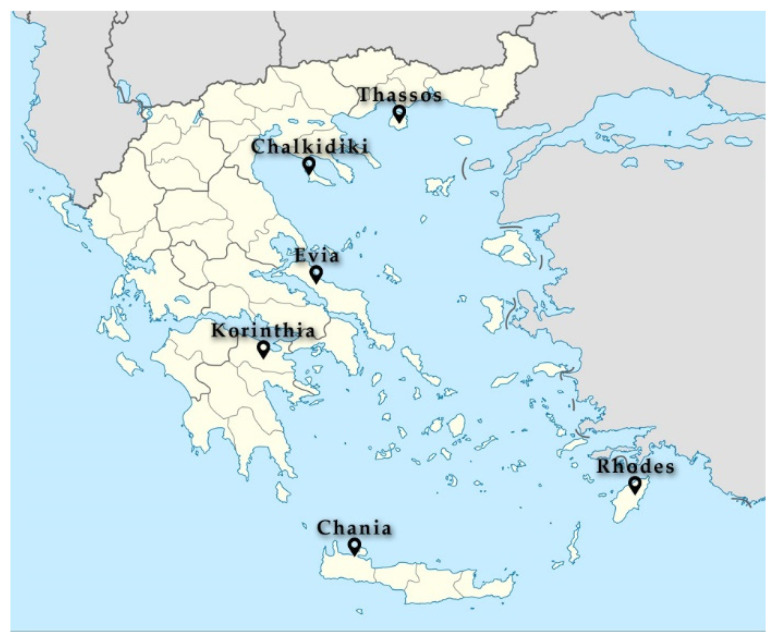
Locations of collected pine honey samples across Greece.

Twenty-seven pine honey samples from diverse locations in Greece (Figure 1) were selected from samples collected in the framework of the national Emblematic Action “The Honeybee Routes”. The classification as pine honey was based on the organoleptic, microscopic (honeydew elements) and electrical conductivity (>0.9 mS/cm) measurement (Table S1, Figure S1).

All samples were stored in glass containers at −18 °C until analysis. Before all assays, the samples were homogenized by stirring thoroughly for at least 3 min. Crystalized samples were liquefied in gentle heat of less than 40 °C for 5 min. Manuka honey UMF 24+ (MGO 1122) (Steens™, New Zealand, LOT 20NZH18) was used as reference honey to compare antibacterial activity of pine honey samples.

### 2.2. Microscopic Examination

Microscopic examination was performed according to von der Ohe et al. [36]. Honeydew elements (HDE) have been counted and the ratio HDE/P (Pollen) is given as the number of honeydew elements over the number of pollen grains from nectariferous plants. Quantitative melissopalynological analysis was based on the method of Yang et al. [37] and results are expressed as total number of all pollen grains (PG) in 10 g honey.

### 2.3. Physicochemical Parameters

All measurements of the physicochemical parameters were performed in duplicate (*n* = 2) unless otherwise stated.

#### 2.3.1. Reagents

Glycerol standard (≥99%) and starch soluble (for analysis) were purchased from Merck (Darmstadt, Germany). Glacial acetic acid (≥99.8%), potassium iodide (≥99.5%), sodium chloride (≥99.5%), sodium hydroxide (≥98%) and buffer solution (pH 4, 7 and 10) were purchased from Honeywell (Charlotte, NC, USA). Sodium acetate trihydrate (99%) was purchased from PENTA chemicals (Prague, Czech Republic) and conductivity standard (1413 μS/cm, 20 °C) was purchased from LLG International (Meckenheim, Germany). Iodine (99.5+) and zinc acetate dihydrate (for analysis) were from Fisher Chemical (Waltham, MA, USA). Potassium hexacyanoferrate (II) trihydrate (98+%) was purchased from Alfa Aesar (Ward Hill, MA, USA). Sodium bisulfite was purchased from Acros Organics (Geel, Antwerp, Belgium).

#### 2.3.2. Moisture Content

Moisture content at 20 °C was determined from the refractive index of honey using a honey refractometer (Hanna, HI, USA). Initially, 2 g of honey was weighed and liquefied in an ultrasonic bath for a few minutes at 35 °C. Then, a small amount of liquefied, homogenized sample was spread on the surface of the prism and we marked the measurement on a percentage scale.

#### 2.3.3. pH and Free Acidity

The determination of pH and free acidity was held by titration to pH 8.3 at 20 °C, according to the ‘Harmonised Methods of the International Honey Commission (IHC)’ [38]. A pH/conductivity device (Hanna, HI 9811-5, USA) was employed. The free acidity of honey measured the content of free acids, expressed in milliequivalents/kg honey (meq/kg).

#### 2.3.4. Electrical Conductivity

Electrical conductivity at 20 °C was determined with the aforementioned portable pH/conductivity device, following the method of the IHC, except for the amount of honey which was reduced to 5 g (from 20 g in the initial method) as it was found to give the same results (data not shown). Results are expressed in mS/cm [38].

#### 2.3.5. Color Analysis

Color measurements were performed using a color photometer (Hanna, HI 96785, USA), that measures the light transmittance of honey compared to analytical-grade glycerol. Honey samples of about 2 g were warmed gently in an ultrasound bath for a few minutes at 35 °C. The liquid honeys without air bubbles were transferred into a plastic cuvette and the color was read. Color grades were expressed in millimeters (mm) Pfund scale [24].

#### 2.3.6. Hydroxymethylfurfural (HMF) after White

For the estimation of HMF content after White, a UV-VIS Spectrophotometer (Shimadzu, UV-1700, Japan) was employed according to the ‘Harmonised Methods of the IHC’ [38]. The method determines the concentration of 5-(hydroxymethyl-)furan-2-carbaldehyde. Results were expressed in milligrams per kilogram (mg/kg).

#### 2.3.7. Diastase Activity (DN) after Schade

The procedure followed was in accordance with the respective method of IHC. For each sample of honey, a repeater was prepared to achieve accuracy in the result and calculate the repeatability (r). The unit of measurement of the enzyme is the unit DN (diastase number) which corresponds to the amount of enzyme needed for breaking down 0.01 g of starch in 1 h at 40 °C [38].

#### 2.3.8. Sugars

For the determination of fructose, glucose and sucrose, a High-Performance Liquid Chromatography with Refractive Index Detector (HPLC-RID) technique was used. The preparation of the samples was achieved according to the literature [38], while for the separation and detection, the following parameters were used: column: Zorbax Carbohydrate Analysis (4.6 mm ID × 150 mm × 5 μm), temperature 35 °C, mobile phase: acetonitrile/water (75/25, *v*/*v*), flow rate: 1.8 mL min^−1^. For the quantification, a five-point calibration curve was created and evaluated for each sugar.

### 2.4. Sensory Evaluation

For the sensory evaluation of samples, a two-round procedure was applied. At the first one the trained testers checked the botanical origin of the sample answering with Yes/No to the question: is the sample pine honey? At the second round, applied only for pine honeys, evaluating the visual, taste and aromatic characteristics, samples were classified in three levels: 3. very good, 2. medium, 1. good evaluated pine honey [39].

### 2.5. Bacterial Strains and Growth Conditions

All tested bacterial strains were isolated, identified and characterized by standard laboratory methods (kindly provided by Professor Spyros Pournaras, School of Medicine, University of Athens, Athens, Greece). Antibacterial activity of pine honeys was tested against *Acinetobacter baumannii*, *Klebsiella pneumoniae*, *Pseudomonas aeruginosa*, *Salmonella* ser. Typhimurium and *Staphylococcus aureus*. The bacteria were routinely grown in Mueller Hinton Broth or Mueller–Hinton Agar (Lab M, Bury, UK).

### 2.6. Determination of Minimum Inhibitory Concentration (MIC)

The determination of minimum inhibitory concentration (MIC) of tested honeys was carried out in sterile 96-well polystyrene microtiter plates (Kisker Biotech GmbH & Co. KG, Steinfurt, Germany) using a spectrophotometric bioassay as described by Tsavea and Mossialos [40]. Approximately 5 × 10^4^ CFUs in 10 μL Mueller–Hinton broth was added to 190 μL of twofold diluted test honey (including manuka honey) at different concentrations which ranged from 25 to 0.78% (*v*/*v*). As control, Mueller–Hinton broth inoculated with bacteria was used. Optical density (OD) was determined at 630 nm using an ELx808 absorbance microplate reader (BioTek Instruments, Inc., Winooski, VT, USA), at t = 0 (prior to incubation) and after 24 h of incubation (t = 24) at 37 °C. The OD for each replicate well at t = 0 was subtracted from the OD of the same replicate well at t = 24. The following formula was used to determine the growth inhibition at each honey dilution: % inhibition = 1 − (OD test well/OD of corresponding control well) ×100. The MIC was determined as the lowest honey concentration which results in 100% growth inhibition [40]. MICs were determined in triplicates in at least two independent experiments.

### 2.7. Determination of Minimum Bactericidal Concentration (MBC)

Minimum bactericidal concentration (MBC) is the lowest concentration of any antibacterial agent that could kill tested bacteria. In order to determine the MBC, a small quantity of sample contained in each replicate well of the microtiter plates was transferred to Mueller–Hinton agar plates by using a microplate replicator (Boekel Scientific, Feasterville, PA, USA). The plates were incubated at 37 °C for 24 h. The MBC was determined as the lowest honey concentration at which no grown colonies were observed [41].

### 2.8. Determination of H_2_O_2_ Accumulation in Honey Samples

The ability to generate H_2_O_2_ in the diluted honey samples was determined using a Megazyme GOX assay kit (Megazyme International Ireland Ltd., Wicklow, Ireland) with some modification [27], which is based on the release of H_2_O_2_ after GOX catalysis of the oxidation of β-d-glucose to d-glucono-δ-lactone. As a standard, 9.8–312.5 μM diluted H_2_O_2_ was used. A total of 40% (*w*/*w*) of the honey solutions in 0.1 M potassium phosphate buffer (pH 7.0) were prepared and incubated for 24 h at 37 °C. Each honey sample and H_2_O_2_ standard were tested in duplicate in a 96-well microplate. The resultant H_2_O_2_ reacts with p-hydroxybenzoic acid and 4-aminoantipyrine in the presence of peroxidase to form a quinoneimine dye complex, which has a strong absorbance at 510 nm. The absorbance of reaction was then measured at 510 nm using a Synergy HT microplate reader (BioTek Instruments, Winooski, VT, USA).

### 2.9. Total Protein Content

Total protein content was measured using Quick Start^TM^ Bradford reagent (Bio-Rad, Hercules, CA, USA) according to manufacturer’s instructions.

### 2.10. Determining the Protein Profile of Honey Samples

For protein determination, 15 μL aliquots of diluted honey samples (50% *w*/*w* in distilled water) were loaded on 12% SDS-PAGE gels [Acrylamide/Bis solution, 37.5:1 (40% *w*/*v*), 2.6% C] and separated using a Mini-Protean II electrophoresis cell (Bio-Rad, Hercules, CA, USA). Protein content was assessed after gel staining with Coomassie Brilliant Blue R-250 (Sigma-Aldrich, Darmstadt, Germany).

### 2.11. Determination of the Antibacterial Activity Due to H_2_O_2_ and Proteinaceous Compounds

The MIC of honey treated with bovine catalase or proteinase K was determined and compared with that of untreated honey [40]. Briefly, 50% (*v*/*v*) honey in Muller–Hinton broth containing 100 μg/mL proteinase K (Blirt, Gdansk, Poland) or 600 U/mL bovine catalase (Serva, Heidelberg, Germany) was incubated for 16 h at 37 °C and then tested after being diluted twofold.

### 2.12. Total Phenolic Content (TPC) and Antioxidant Activity

#### 2.12.1. Reagents

All the solvents used were of analytical grade. Methanol was purchased from Sigma-Aldrich Co (St. Louis, MO, USA). Sodium carbonate, L-ascorbic acid, 2,2-diphenylpicrylhydrazyl (DPPH.), 2,4,6-tris(2-pyridyl)-s-triazine (TPTZ), gallic acid (97.5%), fructose (>99.5%), glucose (>99.5%), maltose monohydrate (>99%), sucrose (>99.5%), Sigma-Aldrich Co (St. Louis, MO, USA). Folin–Ciocalteu (2N) phenol reagent, Iron (III) chloride hexahydrate and Iron (II) Sulfate heptahydrate (FeSO_4_·7H_2_O) were from Honeywell-Fluka (Harvey St., Muskegon, MI, USA).

#### 2.12.2. Sample Preparation

Five grams of honey was dissolved in 50 mL of distilled water. To eliminate the interferences of reducing sugars, the blank measurements were performed using an artificial honey containing: 40% fructose, 30% glucose, 8% maltose, 2% sucrose and 20% water [42]. All measurements were performed in triplicate (*n* = 3).

#### 2.12.3. Total Phenolic Content (TPC)

The total phenol content was determined by a modified colorimetric assay using the Folin–Ciocalteu reagent [43,44]. Briefly, honey samples were dissolved in distilled water (0.1 g/mL), until a clear solution was obtained. Then, 100 µL of the honey solution was added to 1000 μL of Folin–Ciocalteu reagent previously diluted 1:10 with distilled water. After one minute, 300 μL of saturated sodium carbonate (Na_2_CO_3_) solution was added. The mixture was vortexed for 2 min, and the content was transferred into a 1.5 mL cuvette; absorbance was determined after one hour of incubation in the dark at 750 nm against a blank solution. A calibration curve was constructed with gallic acid (0.02–0.2 mg/mL) and the results were expressed as mg of gallic acid equivalents (GAE) per kg of honey.

#### 2.12.4. Ferric Reducing Antioxidant Power (FRAP)

The reducing capacity of honey was estimated by the FRAP assay. This method involves the reduction of the ferric 2,4,6-tripyridyl-s-triazine complex (Fe^+3^-TPTZ) to its ferrous, colored form (Fe^+2^-TPTZ) in the presence of antioxidants [45]. The FRAP reagent consists of 2.5 mL of 10 mM of TPTZ (2,4,6-tripyridyl-s-triazine) solution in 40 mM HCl, 2.5 mL of 20 mM of FeCl_3_ and 25 mL of 0.3 mM of acetate buffer, pH 3.6. Aliquots of 100 μL of the honey solution (0.1 g/mL) were mixed with 900 μL of FRAP reagent and the absorbance of the reaction mixture was measured at 593 nm after incubation at 37 °C for 30 min. A standard aqueous solution of FeSO_4_-7H_2_O (0.05–0.5 mM) was used for the construction of the calibration curve and the results were expressed as mmol Fe^+2^/Kg of honey.

#### 2.12.5. Antiradical Activity (DPPH)

The radical scavenging activity of honey was estimated spectrophotometrically using the stable free radical 2,2-diphenyl-1-picryl-hydrazile (DPPH). The principle of the method is based on the discoloration (purple) of DPPH solution in the presence of an antioxidant [46]. In short, 0.75 mL of DPPH solution (136 μM) was mixed with 0.375 mL of the honey solution (0.1 g/mL). The mixture was shaken vigorously and then incubated for 60 min at 25 °C in the dark; the absorbance of the remaining DPPH was determined at 517 nm against a blank solution. The scavenging activity was expressed as a percentage of absorbance reduction (RSA%) according to the following equation: RSA % = [(A_t = 0_ − A_t = 60_)/A_t = 0_] × 100, where A_t = 0_ is the absorbance of the solution at t = 0 min and A_t = 60_ is the absorbance of the DPPH solution after 60 min of incubation [47]. All measurements of standards and samples were performed in triplicate.

### 2.13. Statistical Analysis

Correlation analysis was conducted using Spearman’s correlation analysis. Data from Table S1 were tested to study any correlation among the variables. Values of *p* < 0.05 indicated statistically significant differences. Statistical analysis was performed using the SPSS version 13.0 statistical package (SPSS, Inc., Chicago, IL, USA).

## 3. Results and Discussion

### 3.1. Microscopic Examination

A significant number of honeydew elements were detected in all honeys. The great majority of the samples also contained fungal spores which are present solely in pine honeys [48]. The HDE/P ratio showed a great variation and was on average 8.23 ± 16.98, while the total number of pollen grains in 10 g of honey was 57 871 ± 55 867 (Table 1 and Table S1). In about half of the samples, the HDE/P ratio was below 3, which is the minimum value for honeydew honeys proposed in the past [49]. However, later studies have shown that the HDE/P ratio of honeydew honeys may vary and Greek pine honeys have relatively few honeydew elements and large numbers of pollen grains [39,48,50,51,52,53]. Thus, the samples were considered typical Greek pine honeys.

### 3.2. Physicochemical Parameters

The average physiochemical parameters of pine samples are presented in Table 1 (full data are available as Supplementary Materials Table S1). Moisture content, pH value and free acidity averaged 16.1 ± 1.0%, 4.7 ± 0.2 and 25.5 ± 4.6 meq/kg, respectively, values in accordance with previous publications regarding pine honey produced in Greece and Turkey [54,55,56,57,58]. Electrical conductivity values ranged between 0.91 and 1.31 mS/cm, averaging 1.11 ± 0.13 mS/cm. Moisture, free acidity, and electrical conductivity values are within legal requirements [6,7] for unifloral Greek pine honey (less than 20%, less than 50 meq/Kg honey and more than 0.9 mS/cm, respectively). Color values averaged 91.9 mm Pfund but presented high variability among samples (SD = 15.9). HMF was as low as 1 ± 1.25 mg/Kg of honey, with seven samples having no HMF at all, with these values being well below the legal limit of 40 mg/Kg. It is known that Greek pine honey has a low tendency to form HMF [59], which may be attributed to the less acidic nature of pine honey compared to other honey varieties. Fallico et al. [60] reported that, among others, pH values were correlated with the formation of HMF in honey and high pH honeys had a lower formation of HMF. Diastase activity averaged 20.9 ± 8.71 DN which is in accordance with previous works with Greek pine honey [56,57], but visibly higher than values reported for Turkish pine honey [55,58].

It should be stated that a certain number of samples showed relatively low diastase activity, two of them being slightly lower than the legislation limit of 8 DN (see Table S1). Honey enzyme is affected by storage and exposure to high temperatures [61]; however, this is not the case here as the samples are fresh. Fluctuation within a certain honey variety of fresh honey samples is known and can be explained by poor processing of nectar by the bees during an abundant nectar flow or seasonal activity of the pharyngeal glands [62], age of the bees, environmental conditions and beekeeping practices [61].

Fructose concentration was higher than glucose, ranging from 25.87 to 39.22%, while glucose from 14.38 to 33.20%. The average value for the sum of fructose and glucose was found to be 57.75 ± 6.15%, characteristic for a honeydew honey, like pine honey is. In all samples this value (min = 45.65%, max 69.76%) covers the legislation level for honeydew honeys “more than 45 g/100 g honey” [6]. Fructose/glucose ratio averaged 1.27 proving that pine honey has a moderate tendency to crystallize [63].

### 3.3. H_2_O_2_ Concentration and Total Protein Content of Pine Honeys

Antibacterial activity of honey is mainly attributed to accumulated H_2_O_2_ in diluted honey. The average value of accumulated H_2_O_2_ in 40% of honey solutions after 24 h was 3341 ± 921 µM and ranged from 1911 to 5620 µM (Table 1). The observed average ability of pine honey samples to generate high levels of H_2_O_2_ is substantially higher than in other types of honeydew honey, whereas total phenolic content was similar in both groups of honeydew honey samples [28]. In our previous study, the average value of H_2_O_2_ of Slovak honeydew honeys and blossom honeys was 1800 and 743 µM, respectively [27,28]. We assume that polyphenolic compounds, including specific flavonoids, contribute to higher levels of H_2_O_2_ by their pro-oxidant activities and/or the ability to act as electron acceptors in enzymatic reactions [28].

Apart from H_2_O_2_, defensin-1, an antibacterial bee-derived peptide found in honey, can contribute to the overall antibacterial activity of honey [30,64]. However, the effective concentration of defensin-1 at MIC of honeydew honeys is rather low and its contribution seems to be significant only in blossom honey [28].

Although the overall protein content can vary from honey to honey, the profile of the honey’s most abundant proteins is similar among natural honey of different botanical and geographical origin. In the present study, the average of total honey protein content was 552 ± 128 µg/g, ranging from 301 to 827 µg/g (Table 1). The SDS-PAGE analysis showed an identical protein pattern among pine honeydew honeys where MRJP1 was the most dominant protein (Figure S2). These observations are in accordance with other studies where MRJP1 protein was found to be the most prominent band in all tested honeys, including medical-grade ones [65,66,67]. In fact, all major proteins identified in honey are of bee origin [66,68] and are secreted by hypopharyngeal glands into the nectar during collection and processing [69]. It has been proposed that some of these bee proteins and peptides including MRJP1 and defensin-1 could be considered as markers of honey quality and authenticity [30,70].

### 3.4. Total Phenolic Content (TPC) and Antioxidant Activity

Antioxidant activity is the outcome of different pathways which are not fully elucidated. Numerous assays are commonly employed to determine the antioxidant effect of a substrate. The lack of validated and standardized antioxidant protocols poses a challenge when authors compare their data [42,71]. Therefore, data regarding the antioxidant activity of pine honey are limited and discrepancies are observed.

In the present work, the total phenolic content was estimated to be 451.38 ± 120.38 mg GAE/kg, which is in line with the majority of previously published data. Cavrar et al. [72] studied pine honeys from northern Turkey and reported TPC values of 496 ± 148 mg GAE/kg. Similarly, Nayik et al. [73] determined the TPC of pine honeys from the Kashmir valley and found values of 598.4 ± 3.3 mg GAE/kg. In another study of Turkish pine honeys, Can et al. [33] found similar TPC values of 614.2 ± 55.9 mg GAE/kg. Higher TPC values were found by Karabagias et al. [74] (1583 ± 338 mg GAE/kg) regarding pine honey from Greece. In the lower range were the values by Ozkok et al. [75], reported for pine honeys from Turkey (155.55 ± 2.04 mg GAE/kg).

The antiradical activity of pine honeys was expressed as the percentage of the absorbance reduction (RSA%) of the stable DPPH radical at 517 nm. The values we measured ranged from 42.43 to 79.33% which are in excellent agreement with previously published data from Ekici et al. [76] (57.49 ± 20.15%) and Nayik et al. [73] (55.37 ± 6.8%). The reducing power of pine honeys was determined with the FRAP reagent and expressed as mmolFe^+2^/kg. The values ranged from 1.87 to 9.43 mmol Fe^+2^/kg, and in general were higher than those reported by Can et al. [33] (1.48 ± 0.83 mmol Fe^+2^/kg).

### 3.5. Correlations of Parameters

Correlations were looked into among physicochemical parameters using Spearman’s rho test and the results are presented in Table S2. Moisture content was negatively correlated with H_2_O_2_ value (r = −0.471, *p* = 0.013). Free acidity and pH were negatively correlated (r = −0.447, *p* = 0.019), which is logical since free acids contribute to a more acidic nature of honey. In addition, free acidity was positively correlated with several parameters, namely color (r = 0.577, *p* = 0.002), protein content (r = 0.556, *p* = 0.003), TPC (r = 0.592, *p* = 0.001), DPPH (r = 0.410, *p* = 0.034) and FRAP (r = 0.493, *p* = 0.009). Color has been found to correlate with TPC and DPPH [77]. Apart from free acidity, color was positively correlated with the electrical conductivity (r = 0.482, *p* = 0.011), which was expected since minerals considerably contribute to the color of honey [78] and has been demonstrated in the literature [79]. In addition, positive correlations of color were found with H_2_O_2_ (r = 0.381, *p* = 0.050), TPC (r = 0.399, *p* = 0.039) and FRAP (r = 0.547, *p* = 0.003). Dark honeys are known to have higher antibacterial potential, which is partly connected to the H_2_O_2_ value [80], yet this was not shown in our work. In addition, phenolic compounds contribute to honey color [81] and FRAP has been related to color intensity [82,83,84]. Diastase activity was negatively related to fructose (r= −0.425, *p* = 0.027) for no apparent reason, and positively to protein content (r = 0.590, *p* = 0.001), which was expected due to the protein nature of enzymes. The HDE/P ratio was negatively correlated with fructose content (r = −0.422, *p* = 0.028) and DPPH (r = −0.417, *p* = 0.030). H_2_O_2_ value was strongly positively correlated with protein content (r = 0.501, *p* = 0.008). Higher amount of protein could relate to a higher amount of glucose oxidase which is involved in the production of hydrogen peroxide. As expected, TPC, DPPH and FRAP showed a very strong positive correlation with each other (r = 0.637 and 0.684, *p* < 0.001). In the literature, correlations among these three parameters depend on the honey type [17,81,85]. These data indicate that the antioxidant activity is mainly attributed to phenolic compounds. FRAP values were positively correlated with fructose/glucose ratio (r = 0.412, *p* = 0.033) and H_2_O_2_ value (r = −0.497, *p* = 0.008). Finally, HMF was not correlated with any other physicochemical parameter.

### 3.6. Antibacterial Activity of Pine Honey

Nosocomial infections are a major cause of high morbidity and mortality both in developing and developed countries. Most common nosocomial pathogens include *A. baumannii, P. aeruginosa*, *S. aureus* and *K. pneumoniae*. Infections caused especially by hypervirulent strains of those pathogens are very difficult to treat due to multidrug resistance. Therefore, alternative therapeutic approaches to combat nosocomial infections are urgently needed [86]. On the other hand, *S.* Typhimurium-related serotypes are implicated in salmonellosis, the second most common gastrointestinal infection in Europe, thus leading to serious public health issues and economic losses in the food industry [87].

It is generally acknowledged that MIC measurement in broth is a more sensitive and quantitatively accurate method to study honey antimicrobial activity in comparison to an agar-well diffusion assay due to slower diffusion rates of active substances in agar [9,33]. Therefore, MICs of honeys were determined in broth using a spectrophotometric-based assay.

Honey samples exerted antibacterial activity against all tested bacterial strains. MIC and MBC values are presented in Table 2. MIC values of tested honeys against *S. aureus* varied from 3.125% (*v*/*v*) to 12.5% (*v*/*v*). Nine honeys exhibited comparable MIC values to manuka honey with an MIC of 3.125% (*v*/*v*). Regarding *K. pneumoniae*, the MIC values of tested honeys varied from 6.25% (*v*/*v*) to 12.5% (*v*/*v*) while manuka’s MIC value has been determined at 6.25% (*v*/*v*). Thirteen honeys demonstrated an MIC value equal to manuka honey. Similarly, for honeys that tested against *A. baumannii* and *S.* Typhimurium, the MIC values varied from 6.25% (*v*/*v*) to 25% (*v*/*v*), whereas the MIC value of manuka honey has been determined at 6.25% (*v*/*v*). Five and twelve honey samples, respectively, demonstrated MIC values equal to manuka honey. Interestingly, MIC values of honeys tested against *P. aeruginosa* ranged from 6.25% (*v*/*v*) to 25% (*v*/*v*) whereas the MIC value of manuka has been determined at 12.5% (*v*/*v*), meaning that two honey samples exerted superior antibacterial activity against this particular pathogen, while twenty-one honeys were comparable to that of manuka.

The variation in MICs could possibly reflect differential bacterial susceptibility due to distinct antibacterial mechanisms. Furthermore, it has been shown that *S. aureus,* a Gram-positive bacterium was, in general, more susceptible to honey and other bee products compared to Gram-negative bacteria [12,88], which is in accordance with the present study. However, in a recent study that tested blossom honeys from the Greek island of Lemnos, it was demonstrated that Gram-positive bacteria were more resistant compared to the Gram-negative bacteria [89].

In order to find out whether honey samples exert bacteriostatic or bactericidal activity, MBC was determined (Table 2). The MBC values of all tested honey, including manuka honey, against all tested bacterial strains were identical to the MIC values, demonstrating that pine honey kills bacteria, not just inhibits their growth.

Overall, the antibacterial activity exerted by pine honeys, especially of those honeys demonstrating superior or comparable activity to manuka, warrants further investigation.

### 3.7. Antibacterial Activity of Pine Honey Could Be Attributed to Multiple Mechanisms

The underlying mechanisms that could contribute to exerted antibacterial activity were further assessed in those honey samples demonstrating comparable or superior antibacterial activity to manuka. In that respect, catalase-treated honeys demonstrated higher MIC values up to 16-fold in some cases (some honeys against *S. aureus*, for instance) compared to untreated samples (Figure S3). Of note, two honey samples tested against *P. aeruginosa* did not demonstrate higher MICs after catalase treatment.

Similarly, after proteinase K treatment, 2 out of 14 honey samples tested against *P. aeruginosa*, 9 out of 12 against *S.* Typhimurium, 10 out of 14 against *S. aureus* and 6 out of 11 against *K. pneumoniae* demonstrated higher MICs up to 4-fold. Surprisingly, no increase in MIC value was observed for all tested honeys against *A. baumannii* after proteinase K treatment, indicating that proteins present in honey that might exert antibacterial activity have no effect on this certain pathogen (Figure S3).

Spearman’s analysis was performed to assess the correlation between the physicochemical parameters and antibacterial activity. It is shown (Table 3) that no correlation between pH, HMF content, H_2_O_2_ concentration and the antibacterial activity was observed. Surprisingly, there is a statistically significant positive correlation of moisture and MIC and MBC values against *K. pneuomoniae*, indicating that moisture negatively affects the antibacterial activity against *K. pneuomoniae*. A statistically significant positive correlation of antibacterial and antioxidant activity was observed for *K. pneumoniae* and *S.* Typhimurium. Interestingly, a statistically significant negative correlation was observed between DN and *S. aureus’s* MIC and MBC values, indicating that higher diastase activity correlates with higher antimicrobial activity against *S. aureus.*

Overall, our data indicate multiple mechanisms of antibacterial activity exerted by pine honey. This is further supported by our recent study, whereas RNA−sequencing analysis revealed that pine honey affected the transcriptomic profile of *P. aeruginosa* by inducing the expression of 189 genes and by suppressing the expression of 274 genes [90]. Pine honey treatment exerted a broad range of action on several pathways and biological processes, including oxidative stress, transmembrane transport and regulation of DNA-templated transcription, two-component regulatory systems, ABC transporters and SOS response. Interestingly, pine honey downregulates key physiological responses in *P. aeruginosa* such as quorum sensing, bacterial chemotaxis and biofilm formation [90].

## 4. Conclusions

In this study, 27 pine honeydew samples showed physicochemical and microscopic characteristics within the legal limits, except for diastase activity, a parameter known to be highly variable depending on many factors. The ability of pine honeydew samples to generate high levels of H_2_O_2_ is substantially higher than in other types of honeydew honey, whereas protein content was similar. Furthermore, due to their high polyphenol content, a strong antioxidant activity of honey samples was demonstrated. In addition, various correlations were identified among these parameters.

The antibacterial activity of pine honeydew honey samples was variable and MICs of honey solutions varied from 3.125 to 25% depending on the pathogen. The breakdown of H_2_O_2_ by catalase treatment into honey solution resulted in a significant decrease in antibacterial activity. Similarly, the digestion of honey proteins by proteinase K resulted in lower antibacterial efficacy among honey samples, again depending on specific bacteria. Interestingly, the antibacterial activity of proteinase K-treated honey samples against *A. baumannii* was not affected at all. Taken together, these observations suggest multiple underlying mechanisms of antibacterial activity of pine honeydew honeys.

## Figures and Tables

**Table 1:** Mean values of physicochemical parameters.

	Mean	SD	Min	Max
Moisture (%)	16.1	1.0	14.0	18.2
pH	4.7	0.2	4.0	5.0
Free Acidity (meq/kg)	25.5	4.6	18	38
Electrical conductivity (mS/cm)	1.11	0.13	0.91	1.36
Color (mm Pfund)	91.9	15.9	60	118
HMF (mg/kg)	1.00	1.25	0	4.42
Diastase activity (DN)	20.90	8.71	6.95	37.29
Fructose (%)	31.81	2.64	25.9	39.2
Glucose (%)	25.94	4.30	14.4	33.2
Fructose + Glucose (%)	57.75	6.15	45.7	69.8
Fructose/Glucose	1.26	0.23	1.02	2.17
HDE/P	8.23	16.98	0.13	83.78
PG/10gr	57,871	55,867	4994	232,443
H_2_O_2_ (μM)	3341	921	1911	5620
Protein (μg/g of honey)	552	128	301	827
TPC (mgGAE/kg)	451.38	120.38	277.07	693.32
FRAP (mmol Fe^+2^/kg)	4.91	1.85	1.87	9.43
DPPH (RSA%) ^1^	60.11	9.21	42.43	79.33

^1^ RSA: Radical Scavenging Activity. SD: Standard Deviation.

**Table 2:** Antibacterial activity of pine honeys (*n* = 27) compared to manuka honey expressed as MIC and MBC values.

Honey Number	*S. aureus*		*A. baumannii*		*K. pneumoniae*		*S.* Typhimurium		*P. aeruginosa*	
	MIC	MBC	MIC	MBC	MIC	MBC	MIC	MBC	MIC	MBC
1	3.125	3.125	12.5	12.5	6.25	6.25	6.25	6.25	25	ND
2	3.125	3.125	12.5	12.5	6.25	6.25	6.25	6.25	25	ND
3	3.125	3.125	12.5	12.5	6.25	6.25	6.25	6.25	12.5	ND
4	3.125	3.125	12.5	12.5	6.25	6.25	6.25	12.5	25	ND
5	6.25	6.25	12.5	12.5	6.25	6.25	12.5	12.5	12.5	ND
6	12.5	12.5	25	25	12.5	12.5	12.5	12.5	12.5	ND
7	6.25	6.25	12.5	12.5	6.25	6.25	6.25	12.5	12.5	ND
8	3.125	3.125	12.5	12.5	6.25	12.5	12.5	12.5	12.5	ND
9	6.25	6.25	12.5	12.5	12.5	12.5	6.25	6.25	12.5	ND
10	6.25	6.25	12.5	12.5	12.5	12.5	12.5	12.5	12.5	ND
11	12.5	12.5	6.25	6.25	6.25	6.25	6.25	6.25	12.5	ND
12	3.125	3.125	25	25	6.25	6.25	6.25	6.25	12.5	ND
13	6.25	6.25	25	25	6.25	6.25	6.25	6.25	12.5	ND
14	6.25	6.25	6.25	6.25	6.25	6.25	12.5	12.5	12.5	ND
15	6.25	6.25	12.5	12.5	12.5	12.5	12.5	12.5	12.5	ND
16	3.125	3.125	6.25	6.25	6.25	6.25	6.25	6.25	6.25	ND
17	6.25	6.25	12.5	12.5	12.5	12.5	12.5	12.5	12.5	ND
18	6.25	6.25	12.5	12.5	12.5	12.5	12.5	12.5	12.5	ND
19	3.125	3.125	12.5	12.5	6.25	6.25	6.25	6.25	6.25	ND
20	6.25	6.25	12.5	12.5	12.5	12.5	12.5	12.5	12.5	ND
21	6.25	6.25	12.5	12.5	12.5	12.5	12.5	12.5	12.5	25
22	12.5	12.5	12.5	12.5	12.5	12.5	12.5	12.5	12.5	12.5
23	12.5	12.5	12.5	12.5	12.5	12.5	12.5	12.5	12.5	12.5
24	3.125	3.125	6.25	6.25	12.5	12.5	25	25	25	25
25	6.25	6.25	12.5	12.5	12.5	12.5	6.25	6.25	12.5	12.5
26	6.25	6.25	12.5	12.5	12.5	12.5	12.5	12.5	12.5	12.5
27	6.25	6.25	6.25	6.25	12.5	12.5	12.5	12.5	12.5	12.5
Manuka	3.125	3.125	6.25	6.25	6.25	6.25	6.25	6.25	12.5	12.5

Values expressed as % (*v*/*v*). ND: Not Determined.

**Table 3:** Correlation coefficient (r) and significance (parenthesis) values calculated by Spearman’s correlation analysis.

	*S. aureus*		*A. baumannii*		*K. pneumoniae*		*S.* Typhimurium		*P. aeruginosa*
	MIC	MBC	MIC	MBC	MIC	MBC	MIC	MBC	MIC
Moisture	0.363	0.363	0.148	0.148	0.481 **	0.408 *	0.371	0.367	0.157
	(0.058)	(0.058)	(0.451)	(0.451)	(0.010)	(0.031)	(0.052)	(0.054)	(0.424)
pH	−0.036	−0.036	0.137	0.137	0.117	0.127	−0.110	−0.213	−0.171
	(0.855)	(0.855)	(0.487)	(0.487)	(0.552)	(0.518)	(0.579)	(0.277)	(0.385)
HMF	−0.092	−0.092	−0.065	−0.065	−0.327	−0.371	−0.345	−0.272	−0.135
	(0.641)	(0.641)	(0.741)	(0.741)	(0.089)	(0.052)	(0.072)	(0.161)	(0.493)
DN	−0.584 **	−0.584 **	−0.097	−0.097	−0.155	−0.161	−0.194	−0.262	−0.010
	(0.001)	(0.001)	(0.624)	(0.624)	(0.431)	(0.414)	(0.324)	(0.178)	(0.960)
H_2_O_2_	−0.242	−0.242	−0.297	−0.297	0.137	0.232	0.155	0.098	−0.216
	(0.215)	(0.215)	(0.125)	(0.125)	(0.486)	(0.234)	(0.430)	(0.620)	(0.269)
Protein	−0.420 *	−0.420 *	−0.197	−0.197	−0.137	−0.089	−0.125	−0.035	−0.174
	(0.026)	(0.026)	(0.316)	(0.316)	(0.486)	(0.651)	(0.528)	(0.861)	(0.377)
TPC	0.051	0.051	−0.285	−0.285	0.288	0.411 *	0.316	0.346	−0.101
	(0.795)	(0.795)	(0.142)	(0.142)	(0.137)	(0.030)	(0.102)	(0.072)	(0.609)
DPPH	0.306	0.306	−0.365	−0.365	0.554 **	0.554 **	0.381 *	0.371	−0.190
	(0.113)	(0.113)	(0.056)	(0.056)	(0.002)	(0.002)	(0.045)	(0.052)	(0.334)
FRAP	0.136	0.136	−0.202	−0.202	0.563 **	0.688 **	0.432 *	0.421 *	−0.216
	(0.490)	(0.490)	(0.302)	(0.302)	(0.002)	(0.000)	(0.022)	(0.026)	(0.270)

* Correlation is statistically significant at the 0.05 level; ** Correlation is statistically significant at the 0.01 level.

## Data Availability

Not applicable.

## Supplementary Materials

The following supporting information can be downloaded at: https://www.mdpi.com/article/10.3390/foods11070943/s1, Table S1: Full data of each tested honey, Table S2: Correlation coefficient (r, first value) and significance (second value) of physicochemical parameters of pine honey calculated by Spearman’s correlation analysis, Figure S1: Sensory three−point scale evaluation of pine honey samples, Figure S2: Protein profile of pine honeydew honey samples (*n* = 27) from Greece, Figure S3: MIC values after proteinase K (MICp) and bovine catalase (MICc) treatment of honey samples.
