# Supplementary material for: Physicochemical Characterization and Biological Properties of Pine Honey Produced across Greece
Source: Foods. 2022 Mar 25;11(7):943. doi: 10.3390/foods11070943 (PMC8997407; doi:10.3390/foods11070943)
Supplement: Supplementary file 1 [file foods-11-00943-s001.zip › foods-1647554-supplementary.pdf]

Table S1. Full data of each honey sample tested in this study

Honey No.	Reference number	Geographical location	Organoleptic	Moisture %	pH	Free Acidity (meq/kg)	Electrical conductivity (mS/cm)	Colour (mm Pfund)	HMF (mg/kg)	Diastase activity (DN)	Fructose (%)	Glucose (%)	Fructose + Glucose (%)	Fructose / Glucose	HDE / P	PG/10g	H_2O_2 (μM)	Total protein content (μg/g)	TPC (mgGAE/kg)	DPPH (RSA%)	FRAP (mmol Fe+2/kg)
1	8	Corinthia	3	16.5	4.7	22	0.95	67	1.79	22.94	30.9	26.6	57.5	1.16	17.30	27129	2430	445.70	380.14	42.43	3.26
2	13	Corinthia	3	15.7	4.8	23	1.00	76	0.37	34.68	31.4	27.5	58.9	1.14	11.29	39636	2467	527.34	296.40	44.44	1.87
3	20	Evia	3	15.5	4.8	26	0.91	93	0.59	25.26	32.1	27.6	59.7	1.16	3.07	55244	4703	655.65	422.07	53.51	2.52
4	26	Evia	3	15.6	4.8	23	0.94	60	0.29	19.98	31.8	29.2	61	1.09	1.23	93471	3118	540.71	387.23	55.15	4.73
5	30	Evia	3	15.5	4.8	22	0.93	64	0.78	16.52	32.2	29.4	61.6	1.10	1.24	53024	2737	463.66	523.18	56.21	3.04
6	50	Rhodes	2	18	4	26	1.22	88	1.12	14.28	29.3	23.9	53.2	1.22	37.31	10260	2285	515.89	411.76	54.79	4.45
7	64	Rhodes	3	16.2	4.5	29	1.27	95	4.42	9.34	32.6	29.3	61.9	1.11	3.29	27283	2664	664.09	510.94	62.64	4.29
8	65	Evia	2	15.5	4.8	29	1.18	116	0.22	19.74	39.2	30.5	69.8	1.28	2.68	32949	4459	628.70	693.32	62.17	9.43
9	74	Thassos	2	15.9	5	24	1.30	115	0.00	26.51	30.6	27.7	58.3	1.10	3.16	74234	3499	572.05	431.05	55.31	4.75
10	79	Evia	2	16.2	4.9	26	1.06	116	0.89	17.91	31.45	27.02	58.5	1.16	10.73	9150	3892	412.93	633.21	62.37	9.16
11	82	Chania	1	14.5	4.6	26	1.00	93	3.29	8.19	34.5	22.3	56.8	1.55	0.13	223195	3220	465.29	382.16	64.69	4.13
12	83	Chania	3	14	5	18	1.26	99	1.04	6.95	32.9	20.1	53	1.64	9.00	11838	2903	368.30	277.07	53.15	3.05
13	88	Thassos	3	16	4.7	24	1.17	87	0.00	33.43	29.5	24.1	53.6	1.22	0.16	232443	2952	722.94	340.14	55.12	3.71
14	91	Thassos	2	17	4.7	24	1.30	104	1.27	18.57	32.5	31.8	64.3	1.02	1.20	55490	1911	486.12	340.36	48.19	3.56
15	93	Thassos	3	16	4.8	24	1.19	103	0.15	26.46	32.3	25.9	58.2	1.25	3.10	33758	3290	611.67	424.42	67.14	5.73
16	95	Chalkidiki	3	14.9	4.9	30	1.14	118	0.44	36.11	25.9	20.6	46.4	1.26	11.28	23148	4049	652.80	488.78	70.21	4.81
17	96	Thassos	3	15.5	4.6	28	1.06	100	0.44	27.86	35.5	29.5	65.1	1.20	1.16	90009	3847	632.94	641.94	65.38	6.88
18	99	Thassos	3	16.5	4.8	24	1.03	90	0,00	24.65	33.9	27.9	61.8	1.22	1.69	30123	5620	563.61	362.29	65.23	5.73
19	100	Chalkidiki	3	15.6	4.8	28	0.99	99	1.57	37.29	29.5	22.7	52.2	1.30	83.78	36500	3908	726.56	376.47	50.84	4.77
20	101	Thassos	3	16.4	4.9	22	1.09	95	1.42	20.68	31.8	25.5	57.3	1.25	1.26	59683	3559	606.18	363.21	60.24	4.96
21	105	Thassos	3	18.2	4.5	24	1.13	83	2.43	17.46	34.9	33.2	68.1	1.05	0.40	56230	2394	538.79	329.31	62.79	4.12
22	109	Evia	2	16.5	5	24	1.36	78	0.00	7.71	32.9	31.8	64.7	1.03	0.99	22566	3316	301.47	450.68	65.70	4.27
23	111	Evia	1	17.5	4.9	18	0.98	67	0.00	8.96	31.8	25.5	57.3	1.25	4.55	15902	3064	310.79	327.16	50.95	3.94
24	112	Thassos	3	16	4.7	26	1.17	90	0.00	26.59	30.2	21.4	51.7	1.41	0.77	88195	4945	695.04	558.04	72.45	5.63
25	116	Thassos	3	16.3	4.9	22	1.00	89	4.15	17.41	31.5	24.2	55.7	1.30	0.39	95166	2482	502.18	618.56	79.33	6.30
26	136	Chalkidiki	3	18.1	4.7	38	1.09	96	0.00	23.57	26.3	20.7	47.0	1.27	2.01	60889	2246	475.55	580.19	65.08	5.33
27	151	Chania	3	15.6	4.6	38	1.23	101	0.46	15.44	31.3	14.4	45.7	2.17	8.93	4994	4252	827.53	637.28	77.49	8.23
		**Mean**	2.63	16.1	4.7	25.5	1.11	91.9	1.00	20.90	31.81	25.94	57.75	1.26	8.23	57871	3341	552.39	451.38	60.11	4.91
		**SD**	0.63	1.0	0.20	4.6	0.13	15.9	1.25	8.71	2.64	4.30	6.15	0.23	16.98	55867	921	127.78	120.38	9.21	1.85
		**Min**	1	14.0	4.0	18	0.91	60	0	6.95	25.9	14.4	45.7	1.02	0.13	4994	1911	301.47	277.07	42.43	1.87
		**Max**	3	18.2	5.0	38	1.36	118	4.42	37.29	39.2	33.2	69.8	2.17	83.78	232443	5620	827,53	693.32	79.33	9.43

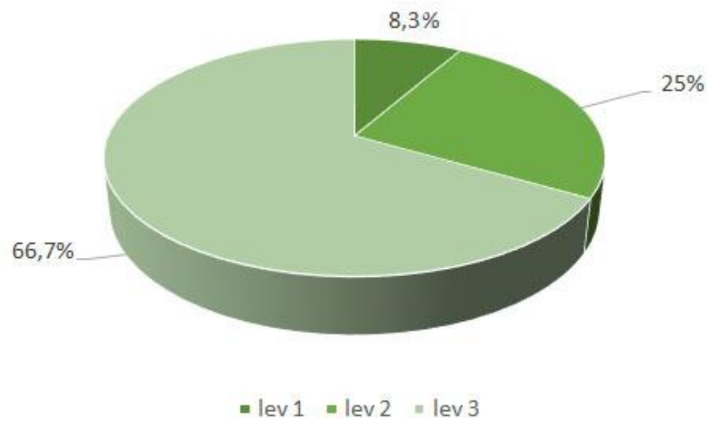

Figure S1. Sensory three-point scale evaluation of pine honey samples.

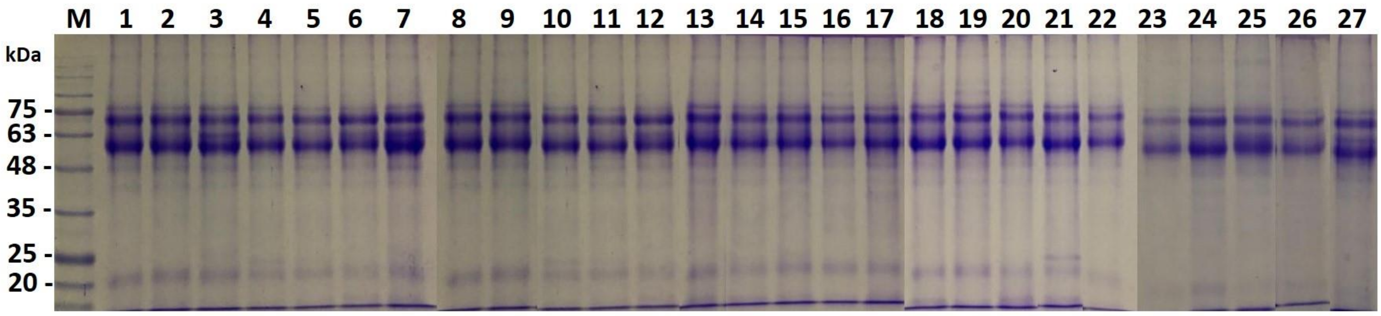

Figure S2. Protein profile of pine honeydew honey samples (n=27) from Greece. A 15 μl of diluted honey samples (50% w/w in distilled water) were separated by 12% SDS-PAGE gels and protein content assessed after gel staining with Coomassie Brilliant Blue R-250. M, protein pre-stained marker.

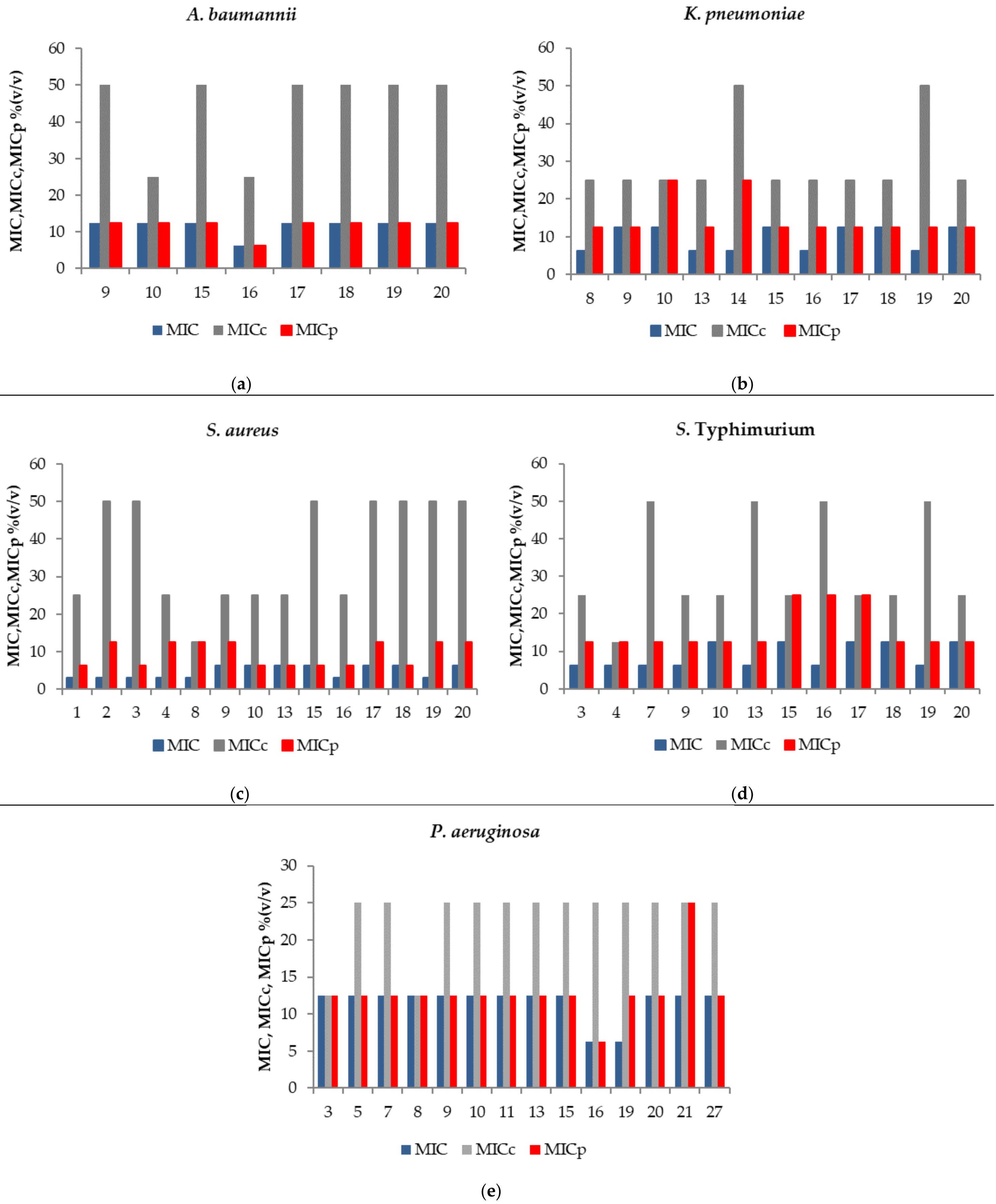

Figure S3. MIC values after proteinase K (MICp) and bovine catalase (MICc) treatment of honey samples tested against *A. baumannii* (**a**), *K. pneumoniae* (**b**), *S. aureus* (**c**), *S. typhimurium* (**d**) and *P. aeruginosa* (**e**). MIC values are expressed as %(v/v).

Table S2. Correlation coefficient (r, first value) and significance (second value) of physicochemical parameters of pine honey calculated by Spearman's correlation analysis.

	Moisture (%)	pH	Free Acidity (meq/kg)	Electrical Conductivity (mS/cm)	Colour (mm Pfund)	HMF (mg/kg)	Diastase (DN)	Fructose (%)	Glucose (%)	Fru + Glu (%)	Fru / Glu	HDE / P	PG / 10gr	H_2O_2 (μM)	Protein (μg/g)	TPC (mg GAE/kg)	DPPH (RSA%)	FRAP (nmol Fe^{+2}/kg)
Moisture	1.000	-0.182	-0.167	0.158	-0.295	-0.039	-0.169	-0.144	0.210	0.108	-0.338	-0.056	-0.102	-0.471*	-0.306	-0.202	-0.049	0.011
	-	0.364	0.404	0.432	0.136	0.845	0.398	0.473	0.293	0.591	0.085	0.782	0.612	0.013	0.120	0.312	0.809	0.955
pH	-0.182	1.000	-0.447*	-0.027	0.126	-0.298	0.013	-0.047	-0.033	-0.044	0.013	0.146	-0.163	0.282	-0.329	-0.085	-0.047	0.018
	0.364	-	0.019	0.893	0.533	0.131	0.947	0.817	0.871	0.828	0.948	0.467	0.418	0.155	0.094	0.673	0.816	0.930
Free Acidity	-0.167	-0.447*	1.000	0.268	0.577**	-0.051	0.241	-0.159	-0.168	-0.149	0.250	0.118	-0.088	0.346	0.556**	0.592**	0.410*	0.493**
	0.404	0.019	-	0.177	0.002	0.802	0.225	0.428	0.403	0.458	0.209	0.558	0.662	0.077	0.003	0.001	0.034	0.009
Electrical Conductivity	0.158	-0.027	0.268	1.000	0.482*	-0.147	-0.204	0.058	0.017	0.003	-0.022	-0.021	-0.240	-0.062	0.113	0.112	0.255	0.204
	0.432	0.893	0.177	-	0.011	0.465	0.308	0.773	0.931	0.989	0.914	0.917	0.228	0.757	0.573	0.579	0.199	0.308
Colour	-0.295	0.126	0.577**	0.482*	1.000	0.009	0.224	-0.005	-0.175	-0.129	0.313	0.225	-0.173	0.381*	0.339	0.399*	0.271	0.547**
	0.136	0.533	0.002	0.011	-	0.963	0.262	0.979	0.381	0.521	0.111	0.259	0.388	0.050	0.084	0.039	0.172	0.003
HMF	-0.039	-0.298	-0.051	-0.147	0.009	1.000	-0.323	0.168	0.008	0.033	0.017	0.088	-0.026	-0.349	-0.063	-0.075	-0.149	-0.164
	0.845	0.131	0.802	0.465	0.963	-	0.100	0.402	0.967	0.872	0.933	0.662	0.896	0.074	0.757	0.710	0.459	0.415
Diastase	-0.169	0.013	0.241	-0.204	0.224	-0.323	1.000	-0.425*	-0.077	-0.134	0.006	0.128	0.357	0.320	0.590**	0.013	-0.047	0.145
	0.398	0.947	0.225	0.308	0.262	0.100	-	0.027	0.702	0.505	0.975	0.526	0.068	0.104	0.001	0.949	0.816	0.471
Fructose	-0.144	-0.047	-0.159	0.058	-0.005	0.168	-0.425*	1.000	0.621**	0.754**	-0.204	-0.422*	0.045	0.082	-0.209	-0.035	0.105	-0.008
	0.473	0.817	0.428	0.773	0.979	0.402	0.027	-	0.001	0.000	0.308	0.028	0.823	0.682	0.296	0.861	0.601	0.967
Glucose	0.210	-0.033	-0.168	0.017	-0.175	0.008	-0.077	0.621**	1.000	0.973**	-0.817**	-0.297	0.119	-0.116	-0.164	0.013	-0.147	-0.135
	0.293	0.871	0.403	0.931	0.381	0.967	0.702	0.001	-	0.000	0.000	0.132	0.555	0.563	0.414	0.947	0.465	0.501
Fru + Glu	0.108	-0.044	-0.149	0.003	-0.129	0.033	-0.134	0.754**	0.973**	1.000	-0.717**	-0.298	0.087	-0.053	-0.177	0.010	-0.124	-0.105
	0.591	0.828	0.458	0.989	0.521	0.872	0.505	0.000	0.000	-	0.000	0.131	0.667	0.792	0.378	0.960	0.538	0.601
Fru / Glu	-0.338	0.013	0.250	-0.022	0.313	0.017	0.006	-0.204	-0.817**	-0.717**	1.000	0.113	-0.064	0.346	0.251	0.176	0.326	0.412*
	0.085	0.948	0.209	0.914	0.111	0.933	0.975	0.308	0.000	0.000	-	0.573	0.753	0.077	0.206	0.379	0.097	0.033
HDE / P	-0.056	0.146	0.118	-0.021	0.225	0.088	0.128	-0.422*	-0.297	-0.298	0.113	1.000	-0.728**	0.027	0.015	-0.087	-0.417*	-0.049
	0.782	0.467	0.558	0.917	0.259	0.662	0.526	0.028	0.132	0.131	0.573	-	0.000	0.892	0.940	0.667	0.030	0.806
PG /10gr	-0.102	-0.163	-0.088	-0.240	-0.173	-0.026	0.357	0.045	0.119	0.087	-0.064	-0.728**	1.000	-0.102	0.219	-0.012	0.101	-0.040
	0.612	0.418	0.662	0.228	0.388	0.896	0.068	0.823	0.555	0.667	0.753	0.000	-	0.613	0.273	0.952	0.617	0.844
H_2O_2	-0.471*	0.282	0.346	-0.062	0.381*	-0.349	0.320	0.082	-0.116	-0.053	0.346	0.027	-0.102	1.000	0.501**	0.341	0.374	0.497
	0.013	0.155	0.077	0.757	0.050	0.074	0.104	0.682	0.563	0.792	0.077	0.892	0.613	-	0.008	0.081	0.055	0.008

Protein	-0.306	-0.329	0.556**	0.113	0.339	-0.063	0.590**	-0.209	-0.164	-0.177	0.251	0.015	0.219	0.501**	1.000	0.235	0.242	0.329
	0.120	0.094	0.003	0.573	0.084	0.757	0.001	0.296	0.414	0.378	0.206	0.940	0.273	0.008	-	0.238	0.223	0.093
TPC	-0.202	-0.085	0.592**	0.112	0.399	-0.075	0.013	-0.035	0.013	0.010	0.176	-0.087	-0.012	0.341	0.235	1.000	0.637**	0.684**
	0.312	0.673	0.001	0.579	0.039	0.710	0.949	0.861	0.947	0.960	0.379	0.667	0.952	0.081	0.238	-	0.000	0.000
DPPH	-0.049	-0.047	0.410*	0.255	0.271	-0.149	-0.047	0.105	-0.147	-0.124	0.326	-0.417*	0.101	0.374	0.242	0.637**	1.000	0.684**
	0.809	0.816	0.034	0.199	0.172	0.459	0.816	0.601	0.465	0.538	0.097	0.030	0.617	0.055	0.223	0.000	-	0.000
FRAP	0.011	0.018	0.493**	0.204	0.547**	-0.164	0.145	-0.008	-0.135	-0.105	0.412*	-0.049	-0.040	0.497**	0.329	0.684**	0.684**	1.000
	0.955	0.930	0.009	0.308	0.003	0.415	0.471	0.967	0.501	0.601	0.033	0.806	0.844	0.008	0.093	0.000	0.000	-

*Correlation is statistically significant at the 0.05 level
**Correlation is statistically significant at the 0.01 level
